# Long-term follow-up of sternoclavicular joint injuries with functional outcome analysis from a single-center study

**DOI:** 10.1007/s00068-025-02964-x

**Published:** 2025-09-03

**Authors:** Julia Elisabeth Lenz, Lisa Klute, Leopold Henssler, Lorenz Huber, Pia Hofstetter, Christian Pfeifer, Volker Alt, Maximilian Kerschbaum

**Affiliations:** 1 Clinic of Trauma Surgery, University Medical Center Regensburg, Franz- Josef-Strauß-Allee 11, 93053 Regensburg, Germany; 2 Department of Internal Medicine I, Gastroenterology, Endocrinology, Infectiology and Rheumatology, University Medical Center Regensburg, Franz-Josef-Strauß-Allee 11, 93053 Regensburg, Germany; 3 Department of Orthopaedic Trauma and Hand Surgery, Innklinikum Altötting, Vinzenz-von-Paul-Str. 10, 84503 Altötting, Germany

**Keywords:** Sternoclavicular joint, Dislocation, Trauma, Long-term outcome, Functional results

## Abstract

**Purpose:**

Sternoclavicular (SC) joint injuries are uncommon, limiting comprehensive insights into their clinical management. This study analyzes long-term outcomes, preferred diagnostic and therapeutic modalities, and variations in treatment approaches and subsequent results.

**Methods:**

A retrospective cohort analysis was conducted on traumatic SC joint injuries treated at a Level-1 Trauma Center between January 2004 and October 2016. Chronic, degenerative, tumor-related cases, incomplete records, and incorrectly coded ICD-10 diagnoses were excluded. Ethical approval was obtained. Patient data on demographics, injury details, diagnostics, and therapeutic interventions were collected. Functional outcomes were assessed using ASES and QuickDASH scores.

**Results:**

Seventeen SC joint injuries were included (14 Allmann type III and 3 Allmann types I–II). Functional outcome data were available for 13 patients (76% response rate) with a mean follow-up of 6.5 ± 4.4 years. Non-surgical approaches were effective for Allmann type I–II injuries. Anterior dislocations yielded good functional outcomes with both conservative and surgical treatment. Posterior dislocations were successfully managed with closed reduction or surgery, resulting in favorable long-term function.

**Conclusion:**

This study, one of the largest long-term follow-ups of SC joint injuries, confirms the rarity of these injuries but demonstrates promising functional outcomes with timely diagnosis and appropriate management.

## Introduction

The sternoclavicular (SC) joint, despite being a critical component in shoulder mechanics, remains inadequately studied, particularly regarding its injuries and pathologies. This is primarily due to the low incidence of SC joint dislocations, which account for only 0.5% of all dislocations [1].

Traumatic injuries to the SC joint are typically caused by high-impact events such as road traffic accidents, sports injuries, or falls [2–5]. The direction of the dislocation, either anterior or posterior, is crucial for determining the appropriate treatment approach [2, 4]. Anterior dislocations are generally considered less severe, whereas posterior dislocations pose significant risks to thoracic structures [6–9].

Allmann’s classification system categorizes SC joint injuries into three types. Type I involves strain of the joint capsule and ligaments, type II is a subluxation with partial tearing of these structures, and type III is a complete dislocation with ligament rupture [10].

Diagnosing SC joint injuries presents challenges due to often inconclusive clinical examinations and radiographs. Advanced imaging techniques, such as computed tomography (CT), magnetic resonance imaging (MRI), and sonography, are essential for accurate diagnosis. Treatment options include both surgical and non-surgical methods, with closed reduction being a common approach [11].

Non-surgical treatments are typically reserved for Allmann type I and II injuries and involve immobilization, early functional treatment, and pain management. For SC joint dislocations, both closed reduction and surgical interventions are viable options [4, 12, 13]. Surgical treatments, classified into six types according to Glass et al., include: Open reduction with internal fixation (ORIF) [14–16], resection arthroplasty [17–21], tendon replacement arthroplasty [22–25], tenodesis [26, 27], suture fixation [28] and Kirschner wire and nail fixation [29–31].

Notably, Kirschner wire and nail fixation, despite its application, carries a risk of implant dislocation and is generally not recommended [32–35].

Given the complexity and potential severity of SC joint injuries, more research is needed to enhance understanding and improve treatment outcomes. Further investigation into the biomechanical properties, diagnostic techniques, and long-term effects of different treatment modalities will aid in developing better management strategies for SC joint injuries.

## Methods

All patients treated for traumatic SC joint injuries at a Level-1 Trauma Center between January 1, 2004 and October 20, 2016, were included retrospectively in the study. Patients with chronic or degenerative SC joint injuries, tumor diseases, incomplete records or incorrectly documented patients were excluded. Retrospective classification of sprains and strains into type I and type II, as per the Allmann classification, proved unfeasible based on patient files. Consequently, this study employs the ICD-10 classification, categorizing cases into ‘Dislocation’ and ‘Sprain/Strain’. For ease of comprehension, the combined group of ‘Sprains and Strains’ will be denoted as ‘Sprains’ for the purpose of this study.

### Ethical approval

(17-595-101) was obtained, and data were collected from the patient information system, including sex, date of birth, date of accident, trauma mechanism, ICD-10 classification, dislocation direction if applicable, associated injuries, diagnostics and therapy performed, postoperative procedure if applicable, follow-up, complications, and time-to-intervention. Furthermore, patients were approached via mail and telephone to evaluate patient-specific outcome scores prospectively (see “Outcome scores”) [36–39]. Our research was conducted in accordance with the Declaration of Helsinki.

### Informed consent

was obtained following verbal explanation. During the telephone interviews, the two self-assessment questionnaires Quick- “Disabilities of the Arm, Shoulder and Hand”-Score (QuickDASH) and American Shoulder and Elbow Surgeons Shoulder Form (ASES) were answered by each patient to determine the functional outcome.

### Outcome scores

The self-assessment questionnaires QuickDASH Score (Disabilities of the Arm, Shoulder and Hand) and ASES Score (American Shoulder and Elbow Surgeons) were used to objectively assess the functional therapy outcome of the patients interviewed. Both scores focus on functional limitations in everyday life in a large part of the questions.

The ASES score includes a detailed pain history, instability history, daily living skills and a free text field for comments [36]. The final score is calculated from the value of the visual analogue scale for pain (VAS Pain) and the cumulative values of the ten questions on abilities in everyday life (Likert scale). The German version of the ASES score used in this study was translated by John et al. and tested for reliability and validity [37].

The QuickDASH Score is the abbreviated form of the DASH self-assessment questionnaire for assessing upper limb function [38]. It includes manual skills, social and occupational limitations, pain, tingling paresthesias and sleep disturbances. The version 2.0 of the DASH translated and validated by Günter Germann and Angela Harth for German-speaking countries was used in this study [39].

### Data analysis

Data collection was performed, and standard descriptive analyses of survey data as well as explorative analysis (Mann-Whitney U test; level of significance *p* = 0.05) were conducted using the SPSS software package version 25 (SPSS Inc, Chicago, Illinois). According to established standardized cut-off-values [40], a QuickDASH score of less than 15 points was considered as “no difficulties with the shoulder”, values between 16 and 40 as “difficulties with preserved function”, and a value above 40 as “loss of shoulder function”. In contrast, there is no official interpretation of the ASES score in literature, apart from standardized values in the normal population [41]. Therefore, the presentation and interpretation of the ASES score was based on comparison with normative values from the general population [41].

## Results

### Patient characteristics

**Fig. 1:**
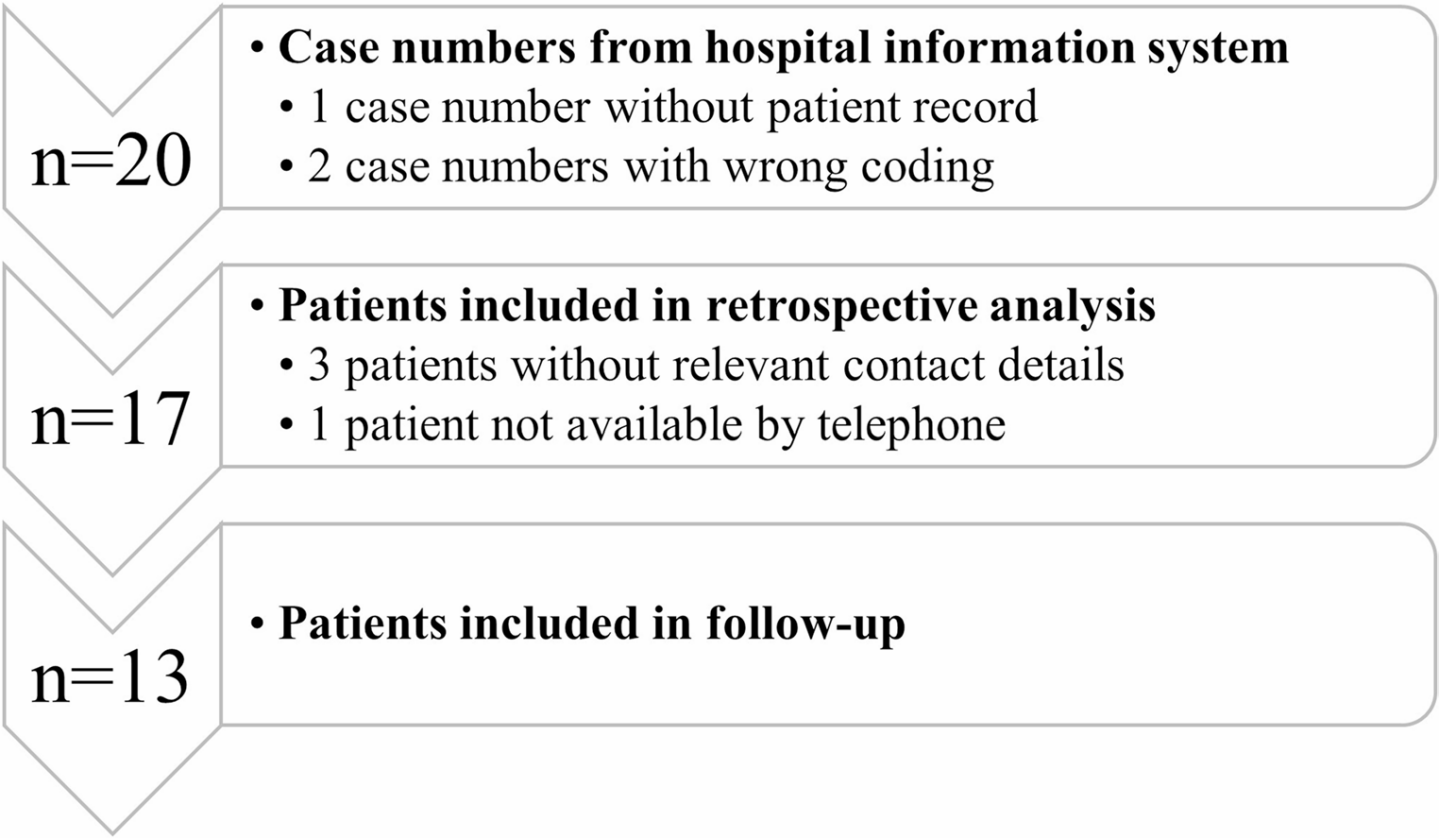
Flow diagram illustrating patient selection for the retrospective study cohort

The search for the ICD-10-GM diagnosis codes S43.2 (Dislocation of the sternoclavicular joint) and S43.6 (Sprain and strain of the sternoclavicular joint) identified 20 cases. Upon application of stringent inclusion and exclusion criteria, our retrospective study cohort comprised 17 patients (see Fig. 1).

Within this cohort, three patients (18%) received a diagnosis of sprain or strain (S43.6), while the remaining 14 (82%) were diagnosed with dislocation (S43.2) based on the ICD-10 classification.

Among the 17 cases, the left side was affected in six patients (35%), the right side was involved in ten (59%). Bilateral involvement was seen in one patient (6%). In instances of clavicular dislocation, anterior displacement occurred in five cases (36%), dorsal in three cases (21%), and lacked documentation in the remaining six cases (43%). The average age at the time of the accident was 35.6 ± 16.7 years, ranging from 16 to 68 years. Mechanisms of injury varied, with falls accounting for four cases (24%), traffic accidents for eight (47%), sporting activities for four (24%), and an atypical incident of catching a box resulting in one injury (6%).

Notably, male patients predominated in SC joint injuries, with six females (35%) and eleven males (65%) among the 17 patients in our study.

### Diagnostic protocol

Among the 17 patients, four (24%) were multiple injured and therefore received a whole body computed tomography (CT) scan, leading to the incidental diagnosis of sternoclavicular (SC) joint dislocation.

Given the non-specific nature of these diagnostic protocols for SC joint injuries, these cases were excluded from further diagnostic tool analysis.

**Fig. 2:**
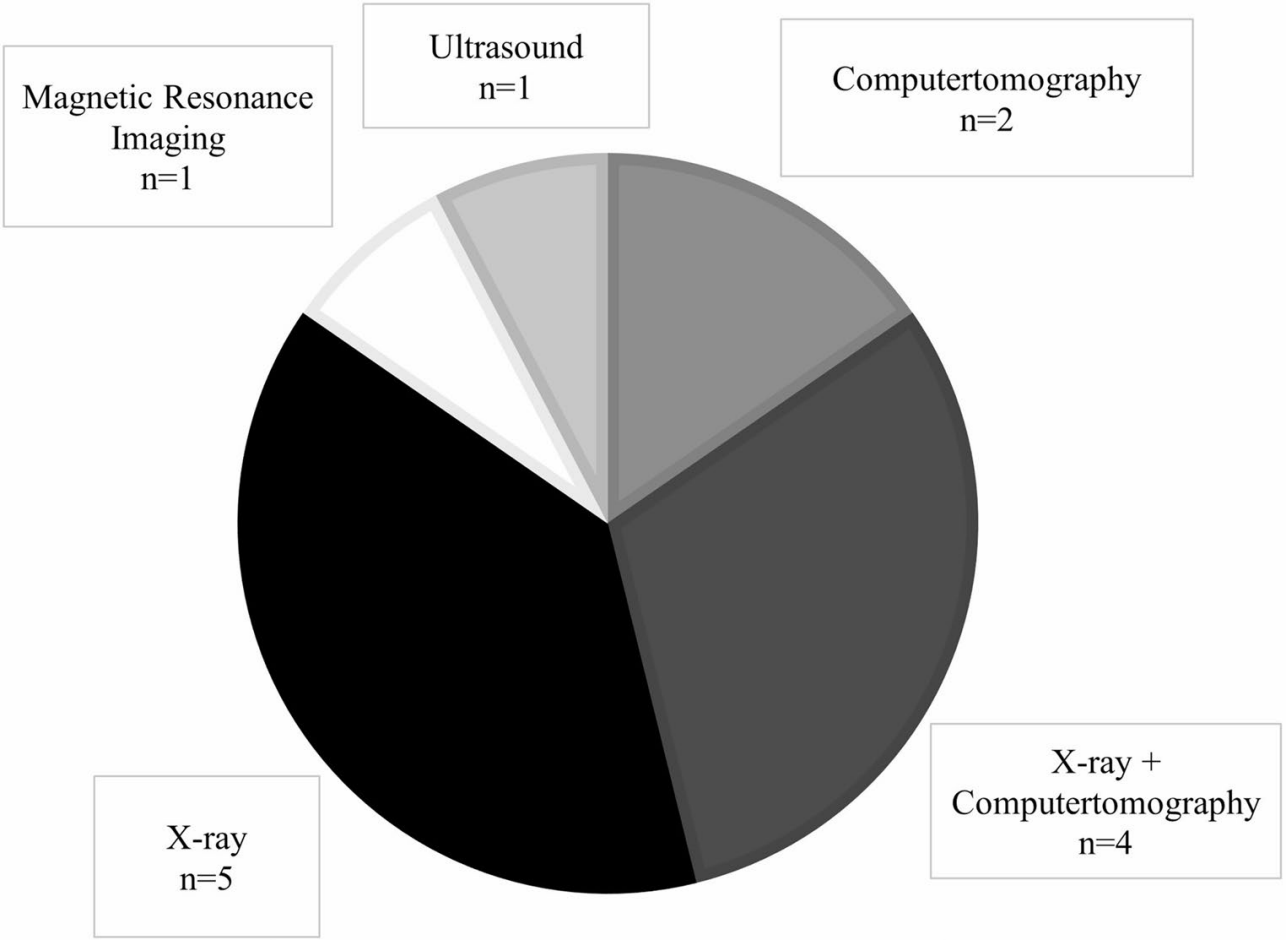
Distribution of imaging modalities used for diagnosis in *n* = 13 patients (excluding polytraumatized patients)

The remaining 13 patients were assessed either by the emergency physician or in the orthopedic surgery consultation. Following initial history-taking and clinical examination of the SC joint, all 13 patients underwent imaging using various modalities, as illustrated in Fig. 2.

Standard X-ray emerged as the method of choice in nine of the 13 cases (69%). Among these, four patients underwent additional CT, while another four opted for alternative modalities such as magnetic resonance imaging (MRI), CT without prior X-ray, or sonography (31% respectively).

### Treatment protocol

Treatment strategies varied, with ten patients managed conservatively (59%), six undergoing surgical interventions (35%), and one successfully undergoing closed reduction (6%). Specifically, all three patients with SC joint sprains were treated conservatively, while among the 14 patients with SC joint dislocation, seven received conservative management (50%), six underwent surgical intervention (42%), and one achieved successful closed reduction (7%) (see table 1).

**Table 1:** Distribution of therapy methods according to injury type

Injury Type	Conservative Therapy	Closed Reduction	Operative Therapy	Total
Sprain	3	0	0	3
Dislocation	7	1	6	14
Total	10	1	6	17

The mean time-to-intervention for the operated patients was 5.1 ± 3.8 days when excluding the outlier with a time-to-intervention of two years. For the conservatively treated patients, it was 2.0 ± 2.3 days on average.

Six patients (35%) in our study underwent surgical intervention, with varying procedures tailored to their specific conditions. Notably, three patients underwent open reduction with plate transfixation (50%). One patient underwent open reduction with tension-band osteosynthesis. In a unique case, the SC joint was openly reduced without fixation following an unsuccessful attempt at closed reduction. Additionally, a patient received treatment involving debridement of the SC joint, followed by resection of the medial clavicle. This procedure was undertaken two years post-trauma.

Non-surgical approaches encompassed immobilization for 2–3 weeks, analgesia, and passive physiotherapy over a 6-week period. In all cases, a mandate for reduced arm weight-bearing persisted for 6 weeks post-trauma, contributing to a comprehensive treatment strategy tailored to each patient’s specific needs.

### Functional outcome

**Fig. 3:**
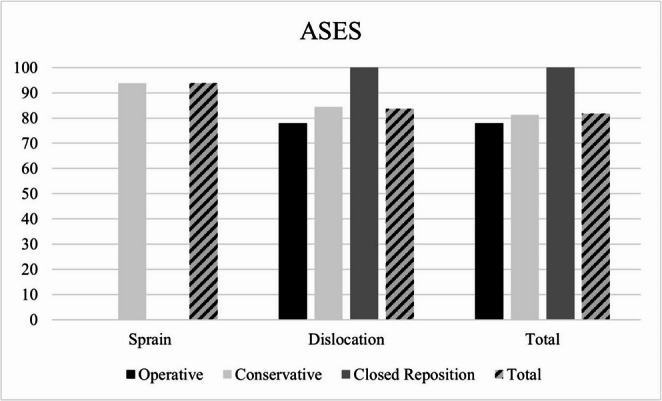
Mean American Shoulder and Elbow Surgeons (ASES) scores according to treatment modality

**Fig. 4:**
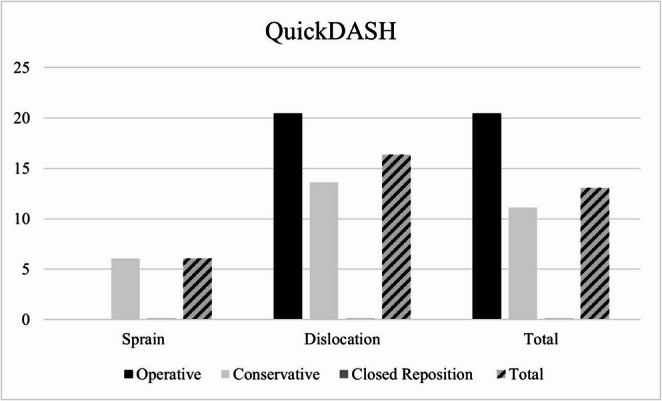
Mean Quick Disabilities of the Arm, Shoulder and Hand (QuickDASH) scores according to treatment modality

Functional outcome could be collected in 13 patients (76% response rate) with a mean follow-up time of 6.5 ± 4.4 years (sprains: *n* = 3; dislocations: *n* = 10). Within the 13 patients, all sprains were treated conservatively, while five patients with dislocations were treated conservatively, one patient received closed reduction and four patients were treated operatively. The mean American Shoulder and Elbow Surgeons (ASES) score for all patients in our study was 85 ± 22 points while the mean QuickDASH score was 14 ± 20 points (see Figs. 3 and 4).

Patients with SC joint sprains showed a tendency to better functional results compared to such with dislocations in both scores (see table 2; *p* = 0.692). Patients with SC joint dislocations treated conservatively resulted in comparable functional outcome scores compared to such treated operatively (see table 2; *p* > 0.05).

**Table 2:** Functional outcomes according to injury and therapy

Pathology	Therapy	*n*	ASES score (mean ± SD)	*p*-value	Quick- DASH (mean ± SD)	*p*-value
Sprains	Conservative Therapy	3	94 ± 9	0,692	6 ± 7	0,692
Dislocations		10	82 ± 24		16 ± 22	
	Conservative Therapy	5	81 ± 18	1,0	16 ± 18	0,9
	Operative Therapy	4	78 ± 33		20 ± 31	
	Closed Reduction	1	100		0	
	Total	13	85 ± 22		14 ± 20	

*ASES* American Shoulder and Elbow Surgeons Shoulder Form, *Quick-DASH *Quick- “Disabilities of the Arm, Shoulder and Hand”-Score

One patient with SC-joint dislocation received closed reduction showing excellent functional outcome (ASES: 100 points; QuickDASH: 0 points). In summary, 77% of SC joint dislocation patients attained an ASES score exceeding 80 points, while three patients fell below this threshold, defining a poor outcome for this study. According to the QuickDASH score, 50% of conservatively treated patients (four out of eight) and 75% of surgically treated patients (three out of four) achieved a satisfactory outcome. Notably, a patient who underwent closed reduction of the SC joint injury achieved an excellent outcome.

### Complications

Complications were observed in five cases within the entire study population (29%). Four complications (67%) occurred in the surgical group, including implant removal due to failure in two patients, shoulder complaints and sensory disturbances in one patient, and post-resection complications in another. Complications were notably less frequent in the conservative treatment group, with one patient (10%) developing a pectoral seroma that required multiple punctures. No patient reported permanent occupational disability.

## Discussion

This study on sternoclavicular (SC) joint injuries included 17 patients. 82% of patients were diagnosed with dislocation and 18% with sprain or strain. The present study population of SC joint injuries is a comparatively large collective compared to the existing literature.

Most injuries occurred due to falls, traffic accidents, sports, or atypical incidents. Within the present study the mean age of patients was 36 years, which is comparable to existing literature [3]. Also, the fact that in our study population SC joint injuries occurred more likely in males is in concordance to the existing literature [3]. In our study collective a statistically higher incidence of dislocations (Allmann type III injury) with 14 cases compared to sprains and strains with three cases were documented. The reason for the relatively high number of type III SC joint injuries in this study population can be attributed to the fact that it is mainly patients with more severe injuries who present to our maximum care hospital. Furthermore, our study’s retrospective design, which excluded cases due to incomplete documentation, may have possibly introduced a selection bias.

Boesmueller et al. observed an opposite distribution of injury types in their study population [3]. Another study also assumes a more frequent occurrence of sprains and strains compared to dislocations [42]. A ratio of anterior to posterior dislocations of 5:3 can be observed in the present study, whereby it should be mentioned that the direction of dislocation was only comprehensible in eight of the 14 SC joint dislocations. In percentage terms, this results in 63% anterior dislocations, which is comparable to the current literature [2, 3, 43].

All 17 patients received imaging. With nine applications, X-ray is thus the most frequently used diagnostic method in this collective. The advantages of X-ray imaging are manifold. However, classic radiographs, such as the chest anterior-posterior projection, are difficult to interpret and not always sufficiently informative due to superimposition of the SC joint with the ribs, spine or sternum [44]. Alternatively, special images, such as the Rockwood image, can be taken to better visualize the SC joint. Ernberg and Potter therefore recommend initial radiographs in cases of suspected SC joint injury, but point out the need for advanced imaging depending on the complexity of the injury [45].

CT is the second most common imaging modality in this study. In the literature, CT is widely considered the gold standard in SC joint diagnosis [11, 12, 46]. Mirza et al. advise that all clinically stable patients with posterior SC joint injury should be examined by computed tomography in order not to miss severe concomitant injuries [47]. Lee and Nasreddine show that based on the CT findings of 38 patients that computed tomographic signs of mediastinal compression are only rarely accompanied by clinical symptoms [33].

With one application each, both magnetic resonance imaging and ultrasound are among the rare diagnostic methods in the present study. This correlates with the experience of other authors, according to which both methods play a subordinate role in the diagnosis of SC joint injuries [11, 26, 48]. Only in pediatric traumatology does it offer the possibility of avoiding radiation exposure [11]. In one case in this study, ultrasound was used for diagnostics. Although ultrasound is not yet used as primary diagnostic procedure, several workgroups have highlighted the potential uprise of the technique in the future [45, 49, 50].

In the treatment of traumatic injuries of the SC joint, non-surgical treatment approach can be followed depending on the classification and the direction of dislocation [32, 35, 51]. In the present study, conservative treatment was performed in a total of ten patients (59%). Three of the ten conservatively treated patients had only sprained their SC joint. The remaining seven patients treated conservatively had a dislocation of the SC joint.

Conservative treatment showed comparable outcomes with fewer complications compared to surgery. SC joint sprains had a tendency of better functional outcomes than dislocations. Notably, 50% of conservatively treated patients and 75% of surgically treated patients achieved satisfactory outcomes. Current literature suggests comparable results. In the study population of Boesmueller et al. the patient group with sprains had an ASES outcome score nine to ten points better than the group with SC joint dislocations [3].

In comparison with other studies, the results of conservatively treated patients in the present study group can be rated as very good [51]. Glass et al. treated 52 patients with SC joint dislocations conservatively [32]. Of these, 68% of the patients had an excellent outcome at follow-up. In contrast to the present study, the authors defined a DASH score of less than 35 as excellent. If, by comparison, a cut-off value of less than 35 points were to be applied to the collective examined in the present study, four out of five patients (80%) are in the excellent range. In our study collective one patient was treated successfully with closed reduction.

Kirby et al. report a success rate of 58.3% (seven out of twelve) in a larger collective of twelve closed reduction attempts [52]. In other studies, higher success rates are observed for closed reduction [7, 32, 34].

Six of our patients with SC joint dislocations were treated surgically, two of them with a dorsal and four with a ventral dislocation. Compared to the non-operatively treated with SC joint dislocations, surgical treatment of SC joint dislocation in our study population showed equivalent results. Our findings are therefor consistent with those of Wang et al., who reported good functional outcomes in 22 patients treated with plate fixation, supporting its role as a viable option in selected cases [53].

While plate fixation was a common method during the study period, recent developments have shifted practice toward soft tissue reconstruction techniques such as tenodesis and non-absorbable sutures, especially for chronic instability. According to El Zouhbi et al., plate fixation remains a valuable option in patients with reduced bone quality [54].

Although specific data on return to work or sports were not uniformly available, no patient reported permanent occupational disability. Gowd et al. highlighted the impact of physical workload, showing that heavy-duty workers may be unable to resume pre-injury activity levels after surgical treatment [55].

While our study on sternoclavicular joint injuries is limited by the low incidence of these rare cases, it serves as a valuable reference point in a field with sparse research. Nevertheless, the small sample size of 17 patients as well as the retrospective character of the study limits the validity of results. Despite these limitations, the study’s clinical relevance is underscored by its focus on a condition with limited existing literature.

The strength of our study lies in its detailed exploration of SC joint injuries, providing a qualitative richness compensating for the limited sample size. The inclusion of diverse treatment modalities and extended follow-up of 6.5 ± 4.4 years enhance the study’s multidimensional perspective. Our findings, while not broadly generalizable, can contribute valuable clinical insights and lay the foundation for future research in this specialized area.

## Conclusion

This study, one of the largest long-term follow-ups of SC joint injuries, confirms the rarity of these injuries but demonstrates promising functional outcomes with timely diagnosis and appropriate management.

## Data Availability

Data is available from the authors upon reasonable request.

## List of abbreviations

ASES: American Shoulder and Elbow Surgeons Shoulder Form
CT: Computed Tomography
MRI: Magnetic resonance imaging
ORIF: Open reduction with internal fixation
QuickDASH: Quick- “Disabilities of the Arm, Shoulder and Hand”-Score
SC joint: Sternoclavicular joint
VAS Pain: Visual analogue scale for pain
